# Accidental Entry of Fish into Throat While Bathing in a Pond

**DOI:** 10.1155/2013/604687

**Published:** 2013-11-25

**Authors:** Pradipta Kumar Parida, Gopalakrishnan Surianarayanan

**Affiliations:** Department of Otorhinolaryngology and Head-Neck Surgery, JIPMER, Puducherry 605006, India

## Abstract

While fish bones are common foreign bodies in the throat, a whole live fish in the pharynx is very rare. We report a case where a whole fish accidentally entered the throat of a 52-year-old male, where it became lodged causing throat pain and dysphagia. The fish was removed as an emergency procedure.

## 1. Introduction

A wide variety of foreign bodies lodging in the upper aerodigestive tract are encountered in otolaryngological practice. Very frequent foreign bodies in children are coins, marbles, buttons, and batteries, whereas in adults common foreign bodies are bones, dentures, and metallic wire. Whole fish is a rare foreign body in the adult age group that has been reported with varied presentation and complications [1, 2]. An animated foreign body can cause mucosal injury due to its movement and secretions. Accidental ingestion and pharyngeal lodging of a whole fish usually occurs while catching fish [1, 3]. Here we present the first case of accidental entry of a live fish into the throat of a man who was bathing in a pond.

## 2. Case Report

**Figure 1:**
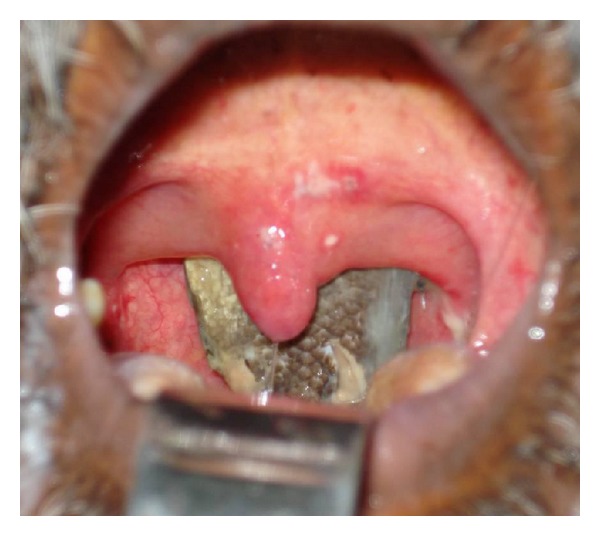
Fish lodged head-up in the patient's oro-/nasopharynx.

A 52-year-old male presented to our outpatient department complaining of the accidental entry of a live fish into his throat, while bathing in a pond, which caused pain and discomfort along with difficulty in swallowing and breathing. Oropharyngeal inspection revealed that the body of the fish was lodged in the oro-/nasopharynx (Figure 1). 

**Figure 2:**
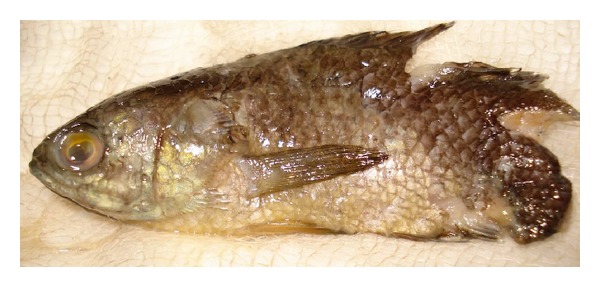
Fish after removal.

An immediate lateral view X-ray of the soft tissues of the neck confirmed that the 10 cm long fish was wedged head-up in the nasopharynx. We removed the fish in one piece (Figure 2) under topical anesthesia (spray) using a tongue depressor and Magill forceps. 

Postremoval control fibroscopic examination of nasopharynx, oropharynx, and laryngopharynx ruled out any residue of fish and mucosal injuries. The postoperative period was uneventful.

## 3. Discussion

Foreign bodies lodged in the upper aerodigestive tract are classified as exogenous and endogenous and as traumatic and atraumatic. The incidence of foreign bodies is the highest in the younger age groups and higher in boys compared to girls. A complete live fish in the aerodigestive tract is a rare finding [3]. The frequent presenting complaints are throat pain and discomfort, dysphagia, respiratory distress, and bleeding [3]. Most of this type of accidents occurs when spearfishers hold the bait fish between their teeth while hunting [1, 3]. Deidiker reported the case of a 45-year-old man who attempted to swallow a whole fish while fishing and drinking with friends who subsequently asphyxiated as a result of upper airway obstruction. In our case a live fish accidentally entered the patient's throat and became lodged head up in his nasopharynx. A complete physical examination and resuscitation are necessary in the initial management. Tracheostomy may be required if stridor is present. Rigid endoscopic removal of foreign body is safe and effective [1]. Control examination of the oral cavity and pharynx including endoscopy is mandatory to rule out any foreign body residua and injuries [3]. A whole fish in throat is an ENT emergency and has to be dealt with swiftly.

## References

[1] Vele DD, Dubey SP (1997). An unusual foreign body: a whole fish in the throat. *Auris Nasus Larynx*.

[2] Deidiker R (2002). Return of the killer fish: accidental choking death on a bluegill (Lepomis macrochirus). *American Journal of Forensic Medicine and Pathology*.

[3] Panigrahi R, Sarangi TR, Behera SK, Biswal RN (2007). Unusual foreign body in throat. *Indian Journal of Otolaryngology and Head and Neck Surgery*.

